# Corrigendum to “Efficient Removal of Brilliant Green Dye Using Mesoporous Attapulgite Clay: Investigating Adsorption Kinetics, Isotherms, and Mechanisms”

**DOI:** 10.1155/tswj/9804935

**Published:** 2025-09-03

A. Al-Jaafari, M. AL-Kazragi, “Efficient Removal of Brilliant Green Dye Using Mesoporous Attapulgite Clay: Investigating Adsorption Kinetics, Isotherms, and Mechanisms,” *The Scientific World Journal* 2024 (2024): 9799127, https://doi.org/10.1155/2024/9799127

In the article, there is an error in the pHpzc (point of zero charge, PZC) of IQATP given in Section 3.4. The correct sentence is shown below:

Furthermore, the pHpzc (point of zero charge, PZC) of IQATP was equal to 5.20, as noted in Figure 8b.

The authors apologize for this error.

